# Metabolic effects of carbon-plated running shoes: a systematic review and meta-analysis

**DOI:** 10.3389/fspor.2025.1710224

**Published:** 2026-01-09

**Authors:** Eiki Nicholas Kobayashi, Rodrigo Ruas Floriano de Toledo, Matheus Oliveira de Almeida, Jan Willem Cerf Sprey, Pedro Baches Jorge

**Affiliations:** 1 Faculdade de Ciências Médicas da Santa Casa de São Paulo, São Paulo, São Paulo, Brazil; 2 Department of Orthopedics and Traumatology, Irmandade da Santa Casa de Misericórdia de São Paulo, São Paulo, São Paulo, Brazil; 3 Health Technology Assessment Unit, Hospital Alemão Oswaldo Cruz, São Paulo, São Paulo, Brazil

**Keywords:** carbon fiber plate, longitudinal bending stiffness, advanced footwear technology, running economy, metabolic cost, oxygen consumption, energetic cost of transport, endurance performance

## Abstract

**Background:**

Advanced footwear technology (AFT) commonly combines compliant, resilient foams with a full-length carbon fiber plate that increases longitudinal bending stiffness (LBS). Whether the plate itself yields metabolic benefits remains debated.

**Objective:**

This study aimed to quantify the effect of carbon plates on metabolic demand during running.

**Methods:**

We conducted a systematic review and meta-analysis of crossover trials comparing plated vs. non-plated running shoes in healthy adults. Databases (MEDLINE, Scopus, LILACS, Embase) were searched in September 2025. Outcomes were running economy (RE) (mL·kg^−1^·km^−1^), metabolic cost (W·kg^−1^), oxygen consumption (mL·kg^−1^·min^−1^), and energetic cost of transport (ECOT) (J·kg^−1^·m^−1^). Random-effects models were used to estimate mean differences (MD).

**Results:**

Fourteen studies met the inclusion criteria. Pooled analyses showed statistically significant reductions favoring plated shoes for RE (MD −5.34 mL·kg^−1^·km^−1^; 95% CI: −8.48 to −2.20), metabolic cost (MD −0.38 W·kg^−1^; 95% CI: −0.59 to −0.16), oxygen consumption (MD −1.23 mL·kg^−1^·min^−1^; 95% CI: −1.82 to −0.63), and ECOT (standardized mean differences −0.37 J·kg^−1^·m^−1^; 95% CI: −0.71 to −0.03). Expressed as percentage change, plated footwear lowered metabolic demand by ∼2%–3% across outcomes (mean −2.75%; range −0.99% to −4.47%). Certainty of evidence was moderate for RE, metabolic cost, and oxygen consumption and low for ECOT (downgraded for indirectness and, for ECOT, imprecision).

**Conclusions:**

In adults, carbon-plated footwear reduces metabolic demand during submaximal running by ≈2%–3%. While concurrent AFT features likely contribute, the pooled evidence supports an association between carbon-plated footwear and reduced metabolic demand, although causality cannot be attributed to the plate alone. Future trials that orthogonally manipulate plate presence and foam properties, while matching mass, stack, and outsole, are needed to isolate plate-specific effects and define plate design parameters that optimize energy transfer across runner body mass.

**Systematic review registration:**

https://www.crd.york.ac.uk/PROSPERO/view/CRD42024520736, PROSPERO CRD42024520736.

## Introduction

From the mid-2010s onward, a new generation of running shoes, advanced footwear technology (AFT), emerged, charecterized by thick, highly resilient midsoles [e.g., polyether block amide (PEBA)-based foams] paired with an embedded stiffening element that increases a shoe's longitudinal bending stiffness (LBS), along with rocker geometries. Together, these features have been associated with improvements in running economy (RE) and metabolic cost across different runner profiles. Recent studies in recreational and mixed samples indicate ∼2%–3% reductions in oxygen cost with AFT, accompanied by changes in ankle mechanics and, in some cases, lower cumulative tibial loading per kilometer (1).

Mechanistically, there is growing consensus that the metabolic benefit arises from the interaction between greater LBS (typically via a curved carbon fiber plate) and the midsole's energy return properties, rather than any single component by itself (2–6). A meta-analysis by Stephen et al. (7) showed that, while the AFT package reduces oxygen consumption and ankle work/power, manipulating only LBS or only energy return does not consistently yield meaningful changes in oxygen consumption, suggesting a synergistic effect between these elements (7).

A comprehensive meta-analysis by Yang et al. (8) synthesized the effects of AFT on running economy, aggregating both plate and foam interventions. While their analysis confirmed an overall metabolic advantage of AFT shoes, it did not isolate the contribution of the carbon fiber plate itself. The present study expands upon Yang et al. (8) by quantifying plate-specific effects across metabolic outcomes using a dataset restricted to plate versus non-plate contrasts.

Regarding relative contributions, recent controlled trials indicate that a curved plate and PEBA foam each tend to deliver gains of similar magnitude in running economy when compared with a control shoe using EVA without a plate. Combining both confers an additional advantage, although smaller than the arithmetic sum of each in isolation (i.e., not fully additive) (9). In parallel, brand and model comparisons demonstrate improvements in the energetic cost of transport (ECOT) among amateur runners of both sexes at training and race paces, supporting generalization beyond elite cohorts (10).

Plate geometry also appears to matter. Recent syntheses suggest curved plates are associated with larger economy gains than flat plates, and experimental data indicate that curvature may modify the forefoot lever and reduce local loads, with potential benefits for performance and injury risk (11). In addition, foam type and wear influence the response: PEBA foams provide an initial advantage over EVA, but that advantage may attenuate with high mileage, indicating that functional durability is relevant to the metabolic effect observed over time (12).

Given the rapid accumulation of evidence and occasional divergence in results, an updated systematic review with meta-analysis is warranted to synthesize the impact of the carbon plate on metabolic outcomes in running—running economy (mL·kg^−1^·km^−1^), metabolic cost (W·kg^−1^), oxygen consumption (mL·kg^−1^·min^−1^), and energetic cost of transport (J·kg^−1^·m^−1^)—compared with non-plated footwear, while isolating, where possible, the plate's effects from other AFT dimensions.

## Methods

### Protocol and registration

This systematic review and meta-analysis were conducted and reported in accordance with the Preferred Reporting Items for Systematic Reviews and Meta-Analyses (PRISMA) and were registered on the International Prospective Register for Systematic Reviews (PROSPERO; CRD42024520736).

### Identification and selection of the studies

MEDLINE via PubMed, Scopus, LILACS, and Embase databases were searched in September 2025. There were no restrictions on publication dates or article language. Search strategies are shown in Table 1. The selection of the studies was conducted on an Excel® (Microsoft Corporation, 1985, 2025, Albuquerque, NM, USA) spreadsheet. An independent reviewer (EK) initially accessed and selected potential studies for inclusion based on title and abstract evaluation. A second independent reviewer (RT) then evaluated and judged the selection of the studies. When necessary, a third reviewer was consulted to solve the discordances. Full texts of selected articles were collected and evaluated in the same manner.

**Table 1:** Database search strategies and total hits.

Databases	PubMed	Scopus	Embase	LILACS
Search strategy	Search: shoes running Sort by: publication date (“shoe s” [All Fields] OR “shoeing” [All Fields] OR “shoes”[MeSH Terms] OR “shoes” All Fields]) AND (“running” [MeSH Terms] OR “running” [All Fields] OR “runnings” All Fields])	TITLE-ABS-KEY (shoes AND running)	“shoes running” OR ((“shoes”/exp OR shoes) AND (“running”/exp OR running)	shoes running AND (db:(“LILACS”)
Total (*n*)	1,312	2,892	1,602	16

Query strings per database (PubMed, Scopus, Embase, LILACS) and the number of records retrieved. See the Methods section for full search dates and inclusion criteria. Abbreviations: *n*, number of records.

There were no restrictions *a priori*; studies meeting criteria were crossover trials. Inclusion criteria were as follows:

Population: healthy adult runners, between 18 and 60 years old (no restriction on performance level).
Intervention: use of carbon-plated shoes
Comparison: carbon-plated shoes versus non-carbon-plated shoes
Outcomes: running economy (mL·kg^−1^·km^−1^) and/or metabolic cost (W·kg^−1^) and/or oxygen consumption (mL·kg^−1^·min^−1^) and/or energetic cost of transport (J·kg^−1^·m^−1^).

### Data extraction

One investigator (EK) extracted data from the selected articles. The following information was recorded: sample size, participant's training level, participant's VO_2máx_, control and intervention shoe's models and shoe's weight, testing speed, type of evaluation (treadmill, track or road), outcome variables (mL·kg^−1^·km^−1^ and/or W·kg^−1^ and/or mL·kg^−1^·min^−1^ and/or J·kg^−1^·m^−1^), and study design. When necessary, authors of the included articles were contacted to request missing data.

### Risk-of-bias assessment

Two independent reviewers (EK and RT) assessed the risk of bias for all included articles. When necessary, a third reviewer was consulted to solve the discordances. Because a tool to assess risk of bias does not exist to evaluate different study designs and biomechanical studies, a modified version of Downs and Black quality index (13) was used, which was implemented in previous systematic reviews (14, 15). Items related to a clinical trial study were disregarded, since the original quality index also evaluates clinical trials. The application of the quality index was made with the same scale and criteria as implemented before in a previous systematic review (15).

The applied scale was composed of 20 items related to information reporting (items 1–9), external validity (items 10 and 11), internal validity (items 12–15), and selection bias (items 16–20). The scores were classified as 0 (high risk of bias) or 1 (low risk of bias). Studies that scored from 0 to 6 were classified as high risk of bias, from 7 to 13 as moderate risk of bias, and from 14 to 20 as low risk of bias (15).

### Certainty of evidence assessment

The certainty of evidence for each primary outcome was evaluated using the Grading of Recommendations Assessment, Development, and Evaluation (GRADE) approach (16). The assessment considered risk of bias, inconsistency, indirectness, imprecision, and potential publication bias across the body of evidence. The overall certainty was rated as high, moderate, low, or very low. Two independent reviewers conducted the GRADE assessment, with disagreements resolved by consensus or consultation with a third reviewer.

### Data analysis

RevMan Web® (Cochrane Collaboration, 2019, 2025, London, UK) software was used for the execution of the meta-analysis, calculation of the heterogeneity (Tau^2^, Chi^2^, and *I*^2^) and overall effects (*Z* and *P* values), and the assembly of forest plot graphics. Data were synthesized using a random-effects model meta-analysis. Due to the methodological heterogeneity of studies, the random-effects model was chosen because it could incorporate statistical heterogeneity. Mean differences (MD), with 95% confidence intervals (CIs), expressed the data of running economy, metabolic cost, and oxygen consumption. Standardized mean differences (SMD) with 95% CI expressed the data of energetic cost of transport, since studies showed different measures for the same parameter.

Results of each meta-analysis were also expressed as percentage of improvement. To facilitate comprehension and observation of the metabolic variations provided by the carbon-plated shoe condition, we synthesized the results of the four meta-analyses with the average percentage of variation between the conditions—carbon-plated shoe vs. non-carbon-plated shoe.

## Results

### Flow of studies through the review

**Figure 1:**
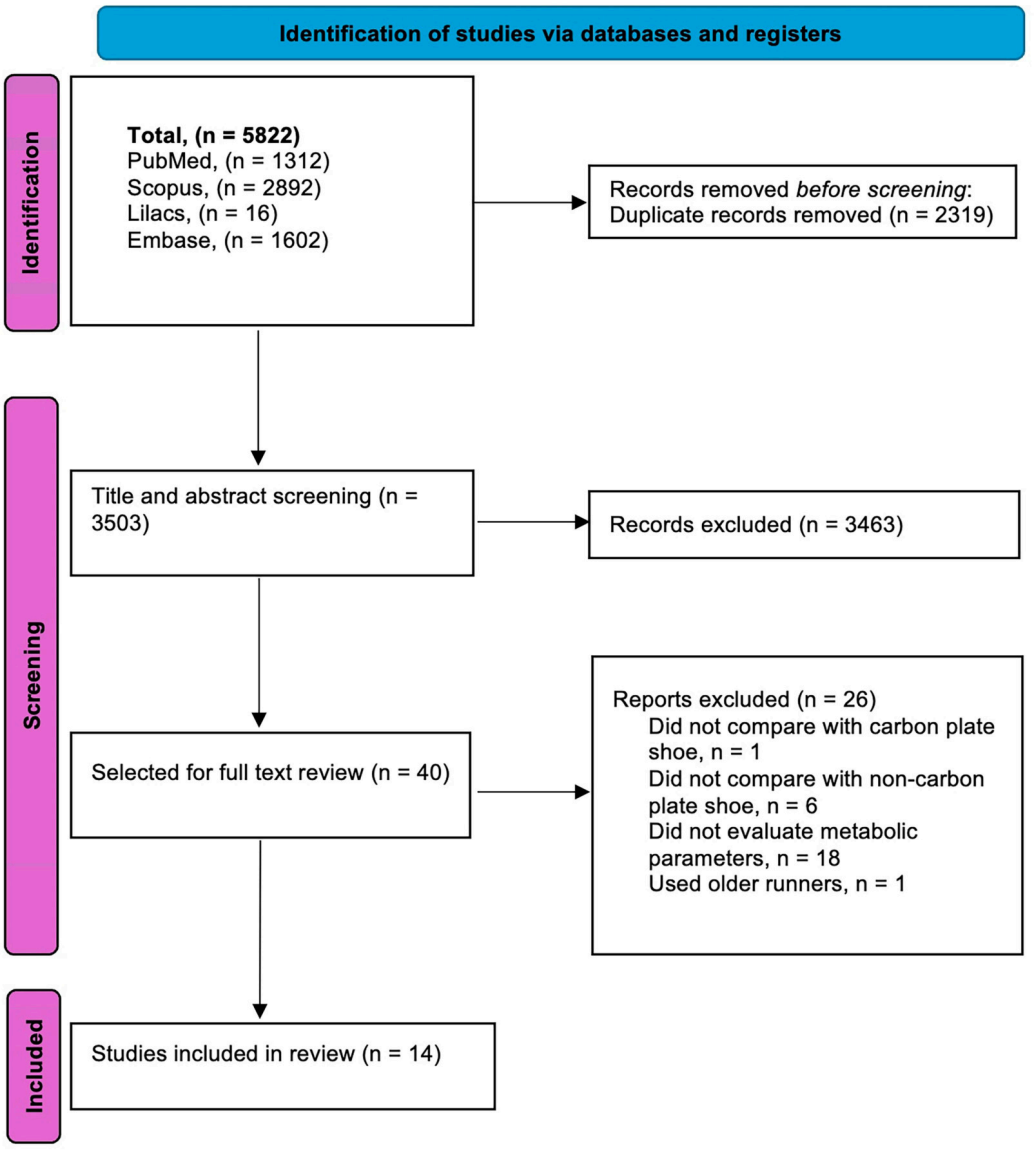
PRISMA flow diagram. Flow of records through identification, screening, eligibility, and inclusion. Database search yielded 5,822 records; 2,319 duplicates were removed; 3,503 records were screened; 40 full texts were assessed for eligibility; 14 studies were included in the qualitative synthesis; 13 contributed data to meta-analyses (one study lacked extractable raw data). Abbreviations: PRISMA, Preferred Reporting Items for Systematic Reviews and Meta-Analyses.

The research in databases (PubMed, Scopus, LILACS, and Embase) identified a total of 5,822 articles, from which 2,319 were duplicates. A total of 3,503 passed the title and abstract screening, and 40 articles were selected for full-text review. Fourteen articles were included in this systematic review. A flow diagram of the full selection process is presented in Figure 1.

### Characteristics of the included articles

The characteristics of the selected studies are shown in Table 2. All 14 studies had a crossover design, from which 8 were randomized and 6 were not randomized, providing data of 271 runners in total. The age of participants from the studies, when they reported, was between 18 and 45 years old. Across studies, participants were predominantly male. Only three studies included female runners, and just one analyzed a female-only sample. Thirteen studies evaluated the runners on a treadmill, of which two used a track besides the treadmill and one evaluated the participants overground. Only one study did not use a treadmill and evaluated the participants on a track and overground.

**Table 2:** Characteristics of included studies.

Study	Sample size (*n*)	Participants’ characteristics	VO_2_max (mL·kg−1·min−1)	Non-carbon-plated shoe	Carbon-plated shoe	Speed	Type of evaluation	Outcomes	Type of study
Joubert and Jones (17)	12 male	High-caliber runners (mean age 26 years)	Not reported	Asics Hyper Speed (227 g)	Nike Vaporfly Next 2% (211 g)	16 km·h^−1^	Treadmill	Metabolic cost (W·kg^−1^); Running economy (mL·kg^−1^·km^−1^); Oxygen consumption (mL·kg^−1^·min^−1^)	Randomized crossover study
Hoogkamer et al. (18)	18 male	High-caliber runners (mean age 23 years)	72.1 ± 3.4	Nike Zoom Streak 6 (250 g)	Nike Vaporfly (250 g)	14 km·h^−1^	Treadmill	Metabolic cost (W·kg^−1^); Running economy (mL·kg^−1^·km^−1^); Oxygen consumption (mL·kg^−1^·min^−1^); Energetic cost of transport (J·kg^−1^·m^−1^)	Crossover study
Nielsen et al. (19)	37 (32 male, 5 female)	Recreational runners (mean age 28 years)	Not reported	Adidas Adizero Adios (190 g)	Nike Vaporfly 4% (210 g)	12.3 km·h^−1^	Treadmill/track	Metabolic cost (W·kg^−1^)	Crossover study
Hunter et al. (20)	18 (10 male, 8 female)	High-caliber runners (age not reported)	Not reported	Saucony Type A (167 g)	Saucony Endorphin Pro (213 g)	13.78 km·h^−1^	Treadmill	Oxygen consumption (mL·kg^−1^·min^−1^)	Crossover study
Perrin et al. (21)	20 male	High-caliber runners (mean age 28 years)	Not reported	Prototype without carbon fiber plate (did not specify weight)	Prototype with carbon fiber plate (did not specify weight)	16 km·h^−1^	Treadmill	Energetic cost of transport (J·kg^−1^·m^−1^)	Randomized crossover study
Flores et al. (22)	11 male	Recreational runners (age not reported)	Not reported	Participants’ own shoes (297 g)	Participants own shoes with a carbon fiber plate (317 g)	11.2 km·h^−1^	Treadmill/overground	Metabolic cost (W·kg^−1^)	Crossover study
Rodrigo-Carranza et al. (23)	28 male	Trained runners (mean age 28 years)	Not reported	Prototype without carbon fiber plate (240 g)	Prototype with carbon fiber plate (248 g)	13 km·h^−1^	Treadmill/track	Metabolic cost (W·kg^−1^)	Randomized crossover study
Hébert-Losier et al. (24)	18 male	Recreational runners (mean age 33 years)	55.8 ± 4.4	Shoes without a carbon fiber plate (313 g)	Nike Vaporfly 4% (211 g)	14.7 km·h^−1^	Treadmill	Metabolic cost (W·kg^−1^); Oxygen consumption (mL·kg^−1^·min^−1^); Energetic cost of transport (J·kg^−1^·m^−1^)	Randomized crossover study
Hunter et al. (25)	19 male	High-caliber runners (mean age 23 years)	Not reported	Nike Zoom Streak 6 (192 g)	Nike Vaporfly 4% (184 g)	15.98 km·h^−1^	Treadmill	Oxygen consumption (mL·kg^−1^·min^−1^)	Randomized crossover study
Martinez et al. (26)	18 female	Competitive runners (between 18 and 45 years)	Not reported	Nike Pegasus 38 (248 g)	Nike Vaporfly Next 2% (176 g)	12.9 km·h^−1^	Treadmill	Metabolic cost (W·kg^−1^); Oxygen consumption (mL·kg^−1^·min^−1^)	Crossover study
Joubert et al. (27)	16 (8 males, 8 females)	Recreational runners (mean age 33 years)	Not reported	Asics Hyper Speed (203.5 g)	Nike Vaporfly Next 2% (187.7 g)	12 km·h^−1^	Treadmill	Metabolic cost (W·kg^−1^); Running economy (mL·kg^−1^·km^−1^); Oxygen consumption (mL·kg^−1^·min^−1^)	Crossover study
Flores et al. (28)	19 male	Recreational runners (mean age 24 years)	Not reported	Prototype without carbon fiber plate (368.9 g)	Prototype with carbon fiber plate (369.6 g)	10.8 km·h^−1^	Overground/track	Energetic cost of transport (J·kg^−1^·m^−1^)	Randomized crossover study
Whiting et al. (29)	16 male	Competitive runners (mean age 27 years)	Not reported	Nike Zoom Streak 6 (196 g)	Nike Vaporfly 4% (203 g)	13 km·h^−1^	Treadmill	Metabolic cost (W·kg^−1^); Running economy (mL·kg^−1^·km^−1^); Oxygen consumption (mL·kg^−1^·min^−1^); Energetic cost of transport (J·kg^−1^·m^−1^)	Randomized crossover study
McLeod et al. (30)	21 male	Experienced runners (mean age 26 years)	Not reported	Prototype without carbon fiber plate (289.25 g)	Prototype with carbon fiber plate (308.75 g)	16 km·h^−1^	Treadmill	Did not provide raw data	Randomized crossover study

Sample size and participant profile, VO_2_max (if reported), comparator and intervention shoes (model/weight), test speed, evaluation setting (treadmill, track, overground), outcomes collected, and study design (randomized/non-randomized crossover). Abbreviations: ECOT, energetic cost of transport; RE, running economy; VO_2_, oxygen consumption. Model and weight of control and carbon-plated shoes are reported as described by the original studies. Most publications did not specify additional AFT features such as midsole foam composition, rocker geometry, or stack height; therefore, these parameters could not be consistently coded. This limits the ability to fully isolate plate effects.

Due to the lack of raw data in McLeod et al.’s study, the authors were contacted, but no response was provided, and the article could not be included in the meta-analyses. In total, 13 studies were included in the meta-analyses.

### Risk-of-bias assessment

The average score for risk-of-bias assessment was 15.7 (0–20 scale). Thirteen studies were classified as low risk of bias and just one study as moderate risk of bias. The items that resulted in the most common higher risk of bias were related to a lack of subjects being representative of the entire population, lack of examiner blinding, lack of adequate adjustment for confounding, and lack of reporting a power calculation (Table 3).

**Table 3:** Methodological quality (Downs and Black index).

Study	1	2	3	4	5	6	7	8	9	10	11	12	13	14	15	16	17	18	19	20	Total
Joubert and Jones (17)	1	1	1	1	1	1	1	0	1	1	0	1	1	1	1	1	1	1	0	0	**16**
Hoogkamer et al. (18)	1	1	1	1	1	1	1	1	1	1	0	0	1	1	1	1	1	0	0	0	**15**
Nielsen et al. (19)	1	1	1	1	1	1	1	1	1	1	0	1	1	1	1	1	1	1	1	1	**19**
Hunter et al. (20)	1	1	1	1	1	1	1	0	1	1	0	0	1	1	1	1	1	0	0	0	**14**
McLeod et al. (30)	1	1	1	1	1	0	1	0	1	1	0	1	1	1	1	1	1	1	0	0	**15**
Perrin et al. (21)	1	1	1	1	1	0	0	0	1	1	0	0	1	1	1	1	1	1	0	0	**13**
Flores et al. (22)	1	1	1	1	1	1	0	1	1	1	0	0	1	1	1	1	1	0	0	1	**15**
Rodrigo-Carranza et al. (23)	1	1	1	1	1	1	1	1	1	1	0	1	1	1	1	1	1	1	0	0	**17**
Hébert-Losier et al. (24)	1	1	1	1	1	1	1	1	1	1	0	1	1	1	1	1	1	1	0	1	**18**
Hunter et al. (31)	1	1	1	1	1	1	1	1	1	1	0	0	1	1	1	1	1	1	0	0	**16**
Martinez et al. (26)	1	1	1	1	1	1	1	0	1	1	0	0	1	1	1	1	1	1	0	1	**16**
Joubert et al. (27)	1	1	1	1	1	1	1	1	1	1	0	0	1	1	1	1	1	0	0	0	**15**
Flores et al. (28)	1	1	1	1	1	1	1	0	1	1	0	0	1	1	1	1	1	1	0	0	**15**
Whiting et al. (29)	1	1	1	1	1	1	1	0	1	1	0	1	1	1	1	1	1	1	0	0	**16**

Item-level scores (0–1) across 20 items grouped as reporting (1–9), external validity (10–11), internal validity (12–15), and selection bias (16–20). Total score 0–20; risk-of-bias categories: 0–6 high, 7–13 moderate, 14–20 low. The mean total score across studies was 15.7; 13 studies were rated low risk, and 1 moderate risk.

### Certainty of evidence assessment (GRADE)

Overall certainty was moderate for running economy, metabolic cost, and oxygen consumption and low for the energetic cost of transport. We downgraded one level for indirectness across outcomes because effects were derived from metabolic surrogates measured predominantly under laboratory conditions. Imprecision was not serious for running economy, metabolic cost, and oxygen consumption (CIs excluded no effect), but serious for the energetic cost of transport (limited information size; CIs near the null). Risk of bias, inconsistency, and publication bias were judged not serious. Details of the GRADE assessment are shown in Table 4.

**Table 4:** Certainty of evidence (GRADE) for primary metabolic outcomes.

Outcome	Running economy (mL·kg^−1^·km^−1^)	Metabolic cost (W·kg^−1^)	Oxygen consumption (mL·kg^−1^·min^−1^)	Energetic cost of transport (J·kg^−1^·m^−1^)
**No. of studies**	4	9	8	5
Study design	Crossover trials (mostly randomized; lab-based)	Crossover trials (mixed randomized/non-randomized)	Crossover trials (mixed randomized/non-randomized)	Crossover trials (mixed randomized/non-randomized)
Risk-of-bias	Not serious	Not serious	Not serious	Not serious
inconsistency	Not serious	Not serious	Not serious	Not serious
Indirectness	Serious (surrogate + lab setting)	Serious (surrogate + lab setting)	Serious (surrogate + lab setting)	Serious (surrogate + lab setting)
Imprecision	Not serious	Not serious	Not serious	Serious (borderline CI; small n)
Publication bias	Undetected (cannot rule out small-study effects)	Undetected (cannot rule out small-study effects)	Undetected (cannot rule out small-study effects)	Undetected (cannot rule out small-study effects)
Overall certainty	⬤⬤⬤○ Moderate	⬤⬤⬤○ Moderate	⬤⬤⬤○ Moderate	⬤⬤○○ Low

Judgments for risk of bias, inconsistency, indirectness, imprecision, and publication bias, with overall certainty per outcome. Indirectness was downgraded one level for all outcomes (metabolic surrogates predominantly measured under laboratory conditions). Imprecision was additionally downgraded for ECOT (limited information size; CI near the null). Symbols: ⬤⬤⬤○ moderate; ⬤⬤○○ low. Abbreviations: ECOT, energetic cost of transport; RE, running economy; VO_2_, oxygen consumption.

### Meta-analysis

**Figure 2:**
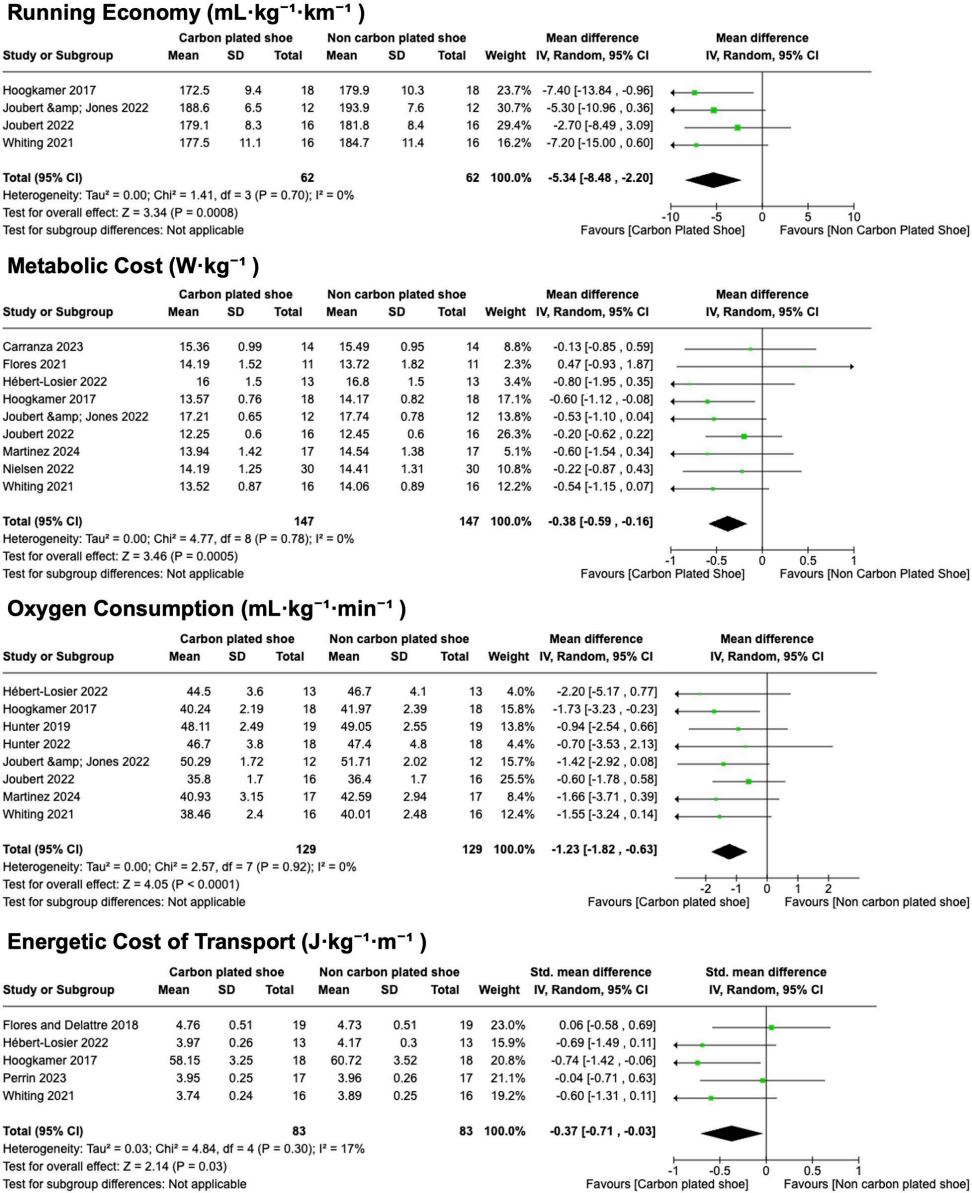
Forest plot of pooled effects for metabolic outcomes. **(A)** Running economy (RE; mL·kg^−1^·km^−1^; *k* = 4): MD −5.34 (95% CI −8.48 to −2.20; *p* = 0.0008). **(B)** Metabolic cost (W·kg^−1^; *k* = 9): MD −0.38 (95% CI −0.59 to −0.16; *p* = 0.0005). **(C)** Oxygen consumption (VO_2_; mL·kg^−1^·min^−1^; *k* = 8): MD −1.23 (95% CI −1.82 to −0.63; *p* < 0.0001). **(D)** Energetic cost of transport (ECOT; J·kg^−1^·m^−1^; *k* = 5): SMD −0.37 (95% CI −0.71 to −0.03; *p* = 0.03). Negative MD values favor the carbon-plate condition (lower metabolic demand). For comparability, when studies reported multiple speeds, we extracted the condition closest to the across-study mean for each outcome (RE 14.14 km·h^−1^; metabolic cost 13.29 km·h^−1^; VO_2_ 13.79 km·h^−1^; ECOT 14.04 km·h^−1^). Abbreviations: CI, confidence interval; MD, mean difference.

As prespecified, we pooled mean differences (MD) with 95% CIs. Negative MD values favor the carbon-plate condition (lower metabolic demand). For comparability across protocols, when multiple speeds were tested within a study, we extracted the condition closest to the across-study mean speed for each outcome (RE, 14.14 km·h^−1^; metabolic cost, 13.29 km·h^−1^; VO_2_, 13.79 km·h^−1^; ECOT, 14.04 km·h^−1^). Forest plots are shown in Figure 2, and percentage changes are summarized in Table 5.

**Table 5:** Percentage change associated with carbon-plate footwear (summary across meta-analyses).

Parameter	MD %	High confidence %	Low confidence %
Running economy	−2.88%	−4.57%	−1.19%
Metabolic cost	−2.64%	−4.09%	−1.11%
Oxygen consumption	−2.84%	−4.20%	−1.46%
Energetic cost of transport	−2.62%	−5.02%	−0.21%
**Mean**	**−2.75%**	**−4.47%**	**−0.99%**

Percentage mean differences (MD%) and 95% CI for each outcome: RE −2.88% (−4.57 to −1.19), metabolic cost −2.64% (−4.09 to −1.11), VO_2_ −2.84% (−4.20 to −1.46), ECOT −2.62% (−5.02 to −0.21). The across-outcome mean change was −2.75% (range −0.99% to −4.47%). Negative values indicate lower metabolic demand with the carbon-plate condition.

Most included studies compared commercially available plated models that also differed in other AFT features, such as midsole foam and geometry. Therefore, the pooled effects represent the typical configuration of modern AFT footwear rather than the isolated contribution of the carbon plate.

**Running economy** (RE; mL·kg^−1^·km^−1^; *k* = 4): Carbon-plate footwear reduced RE (MD −5.34, 95% CI: −8.48 to −2.20; *p* = 0.0008), corresponding to −2.88% (95% CI: −4.57% to −1.19%).

**Metabolic cost** (W·kg^−1^; *k* = 9): Carbon-plate footwear lowered metabolic cost (MD −0.38, 95% CI: −0.59 to −0.16; *p* = 0.0005), i.e., −2.64% (95% CI: −4.09% to −1.11%).

**Oxygen consumption** (VO_2_; mL·kg^−1^·min^−1^; *k* = 8): VO_2_ was lower with carbon-plate footwear (MD −1.23, 95% CI: −1.82 to −0.63; *p* < 0.0001), equivalent to −2.84% (95% CI: −4.20% to −1.46%).

**Energetic cost of transport** (ECOT; J·kg^−1^·m^−1^; k = 5): ECOT showed a smaller yet significant reduction (SMD −0.37, 95% CI: −0.71 to −0.03; *p* = 0.03), or −2.62% (95% CI: −5.02% to −0.21%).

Study-level estimates and heterogeneity statistics are provided in Figure 2 and Table 4.

## Discussion

Across all outcomes, footwear containing a full-length carbon plate demonstrated small-to-moderate improvements in metabolic surrogates of endurance performance compared with non-plated models. Reductions in running economy, metabolic cost, oxygen consumption, and energetic cost of transport were statistically significant in pooled analyses and directionally consistent with the concept that increased longitudinal bending stiffness (LBS) can confer metabolic savings. Although many of the included comparisons contrasted “plate + other AFT features” against “no plate,” several prototype studies held midsole material constant, supporting the inference that the plate itself contributes to the observed savings even when energy return properties are controlled (7, 9, 10).

A central challenge in interpreting these results lies in distinguishing the independent contribution of the plate from that of other co-occurring design elements, such as high-return foams, rocker geometries, increased stack height, and variations in shoe mass. Evidence from controlled experiments indicates that both LBS (mediated by the plate) and midsole energy return influence metabolic cost; however, neither factor alone fully explains the performance improvements seen in modern AFT shoes. When midsole material and geometry were matched—as in prototype studies—plate insertion generally favored lower metabolic demand, suggesting an LBS-specific component. Conversely, comparisons between plated super-foam models and non-plated EVA controls likely overestimate the plate's standalone effect, as these designs inherently differ in foam compliance and geometry (12, 32).

When analyzed by outcome, the pooled mean difference in running economy (−5.34 mL·kg^−1^·km^−1^; approximately equal to −2.9%) supports a plate-related metabolic advantage. Some of the heterogeneity likely reflects differences in testing speed, since stiffer setups tend to perform better at higher velocities, and in the characteristics of the control footwear, yet the favorable direction persists even in plate-only prototype contrasts. For metabolic cost and oxygen consumption, both outcomes decreased under the plated condition. Because increased shoe mass modestly elevates energy cost (∼1% per +100 g), part of the observed benefit may be confounded when the plated model is also lighter; nonetheless, studies that tightly matched shoe mass still demonstrated a plate-related advantage. For the energetic cost of transport, effects were smaller and less precise. ECOT estimates appear particularly sensitive to plate geometry and placement, as comparisons of curved versus flat plates, or of different embedding positions (e.g., under the insole versus within the midsole), indicate that how stiffness is implemented directly influences the metabolic response.

Mechanistically, the plate appears to act through several pathways: by increasing LBS and altering metatarsophalangeal (MTP) joint mechanics and ankle energetics (33); by interacting with the midsole to promote a rocking-lever mechanism (the “teeter-totter effect”) that may reduce distal work (34); and by redistributing joint work without necessarily increasing overall muscle activation (2–6, 33). Experimental manipulations of plate curvature and placement suggest that architecture, rather than mere presence, determines the magnitude of the metabolic benefit. When foam and geometry are held constant, plate insertion still trends toward lower metabolic cost; when combined with compliant, resilient foams, the effect typically becomes larger, reflecting a synergistic rather than purely additive relationship.

Overall, the mean metabolic saving of approximately 2.75% aligns with prior modeling that links a 2%–3% reduction in energetic cost to an expected performance improvement of roughly 1% in marathon times (33, 35). However, since most comparisons contrasted plated AFT models against conventional non-plated controls, translating these laboratory savings directly into race outcomes likely overstates the plate's isolated contribution. The most defensible interpretation is that the carbon plate accounts for a meaningful portion of the observed metabolic gains in modern racing shoes, with the remainder attributable to foam composition, geometry, and other AFT-related design elements.

This review presents several methodological and conceptual strengths. By focusing specifically on plate-versus-non-plate contrasts across multiple metabolic outcomes, it isolates the contribution of longitudinal bending stiffness more precisely than previous syntheses. The inclusion of prototype studies that controlled for midsole material further strengthens the mechanistic interpretation, and the consistent direction of effects across different running speeds and performance levels reinforces the robustness of the findings.

Nonetheless, certain limitations must be acknowledged. Methodological heterogeneity among studies, particularly variations in shoe mass, testing speed, and the reporting of VO_2_max or foot-strike pattern, introduces residual variability that may influence pooled estimates. Because most experiments were conducted in controlled laboratory settings, ecological validity remains limited. Future research should extend beyond controlled laboratory environments by incorporating longitudinal, real-world race data to validate these metabolic findings under competitive conditions. Integrating field measures such as GPS-derived pace stability, finish time, and perceived exertion with pre-race laboratory assessments would allow testing whether the observed ≈2%–3% metabolic savings translate into meaningful performance improvements. Such approaches would strengthen external validity and bridge the gap between laboratory surrogates and race performance.

Few studies stratified their analyses by plate architecture (e.g., curvature, thickness, and taper) or placement, and most failed to report additional AFT parameters such as midsole composition, rocker geometry, or stack height beyond model and weight. The absence of these data prevented systematic control for confounding design factors, thereby constraining the ability to attribute observed metabolic differences exclusively to the plate. Collectively, these aspects restrict generalizability and underscore the need for standardized methodological reporting in future investigations.

An additional limitation concerns participant representation. Most studies enrolled predominantly male runners, with minimal inclusion of female participants. This sex imbalance limits the applicability of the pooled estimates, particularly in light of emerging evidence suggesting that women may have benefited proportionally more from AFT adoption in competitive contexts (36). Future research should ensure sex-balanced sampling and explore whether differences in body mass, biomechanics, and mechanical loading alter the interaction between the plate and other shoe features.

### Future directions—addressing sex and design bias for attribution

Future research should adopt factorial designs that fully cross plate presence (yes/no) with midsole foam type (low vs. high energy return) while tightly matching shoe mass (±10 g), stack height, and outsole construction. A 2 × 2 factorial design would enable orthogonal manipulation of these variables to quantify: (a) the independent metabolic effect of the carbon plate, (b) the independent contribution of foam energy return, and (c) their non-additive synergy. Rigorous mass matching within ±10 g is essential, as even small differences can alter metabolic cost by approximately 1%. Standardized running speeds spanning sub-lactate to near-race paces, prespecified outcomes (running economy, metabolic cost, VO_2_, ECOT), and transparent sample size calculations will enhance reproducibility.

Given that plate efficiency likely scales with vertical load, studies should standardize participant body mass, either by recruiting within narrow weight bands or by prespecified stratification, and perform mass-normalized analyses (e.g., stiffness or deformation relative to body weight). Reporting of plate geometry metrics (curvature radius, thickness, taper, and placement relative to the metatarsal heads) should become routine, and interaction effects (plate × foam × body mass) should be explicitly tested. To improve comparability and reproducibility, future studies should also adopt a standardized reporting framework for carbon-plate design, including longitudinal bending stiffness (N·m·deg^−1^), curvature radius, thickness gradient, vertical placement, and carbon fiber modulus. Establishing such a framework would facilitate cross-study synthesis and clarify how design characteristics, rather than mere plate presence, influence metabolic outcomes.

Emerging evidence suggests that body mass moderates the metabolic response to longitudinal bending stiffness. Accordingly, future trials should identify sex- and mass-specific design targets for carbon plates to optimize energy transfer during running. The optimal combination of stiffness, curvature, taper, and placement likely scales with runner mass and speed, such that body weight-normalized stiffness maximizes ankle and MTP positive work while minimizing distal negative work. Standardizing these analyses will enable the development of individualized “operating zones” describing the conditions under which plate efficiency is greatest.

Although the present meta-analysis could not stratify results by body mass due to the absence of individual participant data, future pooled analyses should predefine narrow body mass bands (e.g., <60 kg, 60–75 kg, and >75 kg) and test for heterogeneity or correlation between body mass and plate-related metabolic savings. This approach would provide an empirical basis for personalized carbon-plate design and directly address the need to define mass-specific parameters that optimize energy transfer efficiency.

## Conclusion

In this systematic review and meta-analysis, carbon-plated running shoes were associated with lower metabolic demand across running economy, metabolic cost, oxygen consumption, and energetic cost of transport, by approximately 2.75%. Certainty was moderate for running economy, metabolic cost, and oxygen consumption, and low for the energetic cost of transport, chiefly due to indirectness (laboratory surrogates) and some imprecision. These findings suggest small but meaningful metabolic savings when running in carbon-plated footwear; however, the specific contribution of the plate cannot be fully disentangled from that of midsole foam and geometry. Future studies should adopt factorial, mass-matched designs, report plate architecture, and test for body mass-dependent responses to quantify the plate-only effect with greater precision.

## Data Availability

The original contributions presented in the study are included in the article/Supplementary Material; further inquiries can be directed to the corresponding author.
